# The Interaction of Vasopressin with Hormones of the Hypothalamo–Pituitary–Adrenal Axis: The Significance for Therapeutic Strategies in Cardiovascular and Metabolic Diseases

**DOI:** 10.3390/ijms25137394

**Published:** 2024-07-05

**Authors:** Ewa Szczepanska-Sadowska, Katarzyna Czarzasta, Wiktor Bogacki-Rychlik, Michał Kowara

**Affiliations:** Department of Experimental and Clinical Physiology, Laboratory of Centre for Preclinical Research, Medical University of Warsaw, 02-097 Warsaw, Poland

**Keywords:** AVP, CRH, ACTH, cardiac failure, glucocorticoids, mineralocorticoids, androgens, estrogens, hypertension, stress

## Abstract

A large body of evidence indicates that vasopressin (AVP) and steroid hormones are frequently secreted together and closely cooperate in the regulation of blood pressure, metabolism, water–electrolyte balance, and behavior, thereby securing survival and the comfort of life. Vasopressin cooperates with hormones of the hypothalamo–pituitary–adrenal axis (HPA) at several levels through regulation of the release of corticotropin-releasing hormone (CRH), adrenocorticotropic hormone (ACTH), and multiple steroid hormones, as well as through interactions with steroids in the target organs. These interactions are facilitated by positive and negative feedback between specific components of the HPA. Altogether, AVP and the HPA cooperate closely as a coordinated functional AVP-HPA system. It has been shown that cooperation between AVP and steroid hormones may be affected by cellular stress combined with hypoxia, and by metabolic, cardiovascular, and respiratory disorders; neurogenic stress; and inflammation. Growing evidence indicates that central and peripheral interactions between AVP and steroid hormones are reprogrammed in cardiovascular and metabolic diseases and that these rearrangements exert either beneficial or harmful effects. The present review highlights specific mechanisms of the interactions between AVP and steroids at cellular and systemic levels and analyses the consequences of the inappropriate cooperation of various components of the AVP-HPA system for the pathogenesis of cardiovascular and metabolic diseases.

## 1. Introduction

**Figure 1:**
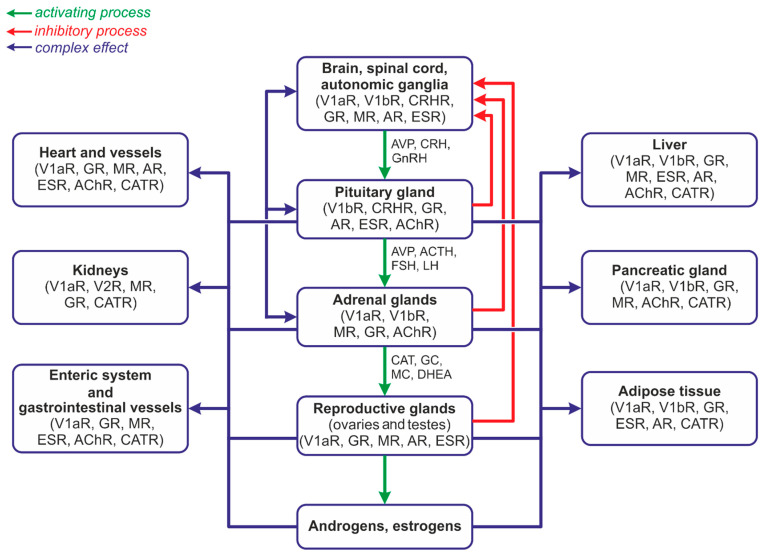
Flow diagram showing the sites of the synthesis, action, and interaction of vasopressin and hormones of the hypothalamo–pituitary–adrenal axis in the central nervous system and peripheral organs. Green arrows—stimulatory effects; red arrows—inhibitory effects. Blue arrows—complex effects (stimulatory or inhibitory, see text for explanations). Abbreviations: AChR—aceytolcholine receptor; ACTH—adrenocorticotropic hormone; AR—androgen receptor; CAT—catecholamine; CATR—catecholamine receptor; CRHR—corticotropin releasing hormone receptor; ESR—estrogen receptor; FSH—follicle-stimulating hormone; GC—glucocorticoid; GnRH—gonadotropin-releasing hormone; GR—glucocorticoid receptor; LH—luteinizing hormone; MC—mineralocorticoid; MR—mineralocorticoid receptor; V1aR—vasopressin V1a receptor; V1bR—vasopressin V1b receptor; V2R—vasopressin V2 receptor.

The first experimental studies showing the close functional relationship between vasopressin and steroid hormones were published over 60 years ago. In 1960 Hilton et al. reported that arterial perfusion of the adrenal glands with arginine vasopressin (AVP) or lysine vasopressin (LVP) potently stimulates the release of cortisol [1]. Subsequently, it has been found that in many instances vasopressin and steroid hormones are secreted together and closely cooperate in the regulation of blood pressure, metabolism, water–electrolyte balance, and behavior in a manner securing survival and the comfort of life. It has also been shown that cellular stress combined with hypoxia, disturbances of metabolism, cardiovascular and respiratory disorders, neurogenic stress, and inflammation may disorganize the cooperation between AVP and steroid hormones. Under homeostatic, unstressed conditions both steroid hormones and vasopressin are released in characteristic diurnal rhythms. The expression of AVP mRNA in the suprachiasmatic nucleus shows a distinct endogenous circadian rhythm. This diurnal rhythm of secretion is also typical for adrenocorticotropic hormone (ACTH) and glucocorticoids (GCs). Interestingly, it appears that the regulation of AVP synthesis by steroids also manifests circadian rhythmicity [2]. Vasopressin interacts with the hypothalamo–pituitary–adrenal axis (HPA) at various levels, i.e., in the hypothalamic nuclei, affecting release of CRH; in the pituitary gland, enhancing ACTH release; and in the adrenal glands, modulating the release or action of multiple steroid hormones (Figure 1). These interactions are facilitated by the positive and negative feedback occurring between specific components of the HPA system with the engagement of other neurotransmitting and neuropeptidergic pathways.

Vasopressin and CRH are synthesized mainly in the hypothalamic nuclei; however, both these peptides can also be produced in other regions of the brain. In the hypothalamus, vasopressin and CRH are released partly by the same cells of the paraventricular nucleus (PVN). Immunostaining studies have shown that virtually all parvocellular CRH neurons in the PVN are stained positively for vasopressin [3] and are co-packaged in neurosecretory vesicles of the hypothalamic–pituitary axons in the median eminence [4]. and in the hypophyseal portal circulation [5,6,7]. There is also evidence that AVP may be necessary for the appropriate action of glucocorticoids, as it was shown that the maximal binding capacities for corticosterone and dexamethasone in the hippocampus and the anterior pituitary are significantly lower in homozygous diabetes insipidus (HDI) rats that do not synthesize vasopressin than in their nondiabetic counterparts. Importantly, the difference could be eliminated by AVP treatment [8]. On the other hand, glucocorticoids were found to exert a negative effect on the release and action of vasopressin. For instance, in rats, corticosterone was found to inhibit the release of AVP from the explants containing a supraoptic nucleus (SON) and sending projections to the neural lobe of the pituitary [9]. Experiments on Sprague Dawley rats have shown that the release of AVP from the hypothalamic slices encompassing PVN and SON neurons could be inhibited by corticosterone, cortisol, testosterone, and 17-beta estradiol in a dose-dependent manner, whereas dexamethasone, aldosterone, and progesterone were not effective [10]. The chronic application of dexamethasone, which is an agonist of the corticosterone receptor, significantly decreased plasma corticosterone and ACTH concentrations and elicited differential changes in the expressions of CRH, ACTH, and AVP in several brain regions, including the cortex, the hippocampus, the hypothalamus, and the cerebellum [11]. There is also evidence that, in the rat, AVP exerts a weak stimulatory effect on the secretion of aldosterone from the glomerulosa cells of the adrenal medulla [12].

It is highly probable that some steroid hormones can modulate vasopressin’s action through actions exerted at the level of the AVP receptors. For instance, experiments on rats have shown that adrenalectomy, which eliminates the main source of circulating steroid hormones, reduces vasopressin V1a receptors (V1aR) in the hippocampus, the dorsolateral septum, and the bed nucleus of the stria terminalis (BNST). Moreover, the effects of adrenalectomy on V1aR in the hippocampus and the BNST can be reduced by treatment with corticosterone and aldosterone [13,14,15]. At the same time, it should be noted that glucocorticoids may act in the opposite way on the expression of V1b vasopressin receptors (V1bR) because the administration of dexamethasone was found to increase V1bR mRNA in the pituitary, whereas adrenalectomy reduced V1bR mRNA. The latter effect could be reversed by the administration of dexamethasone [16].

It appears that negative feedback between glucocorticoids and vasopressin arises in early life. In the rat, applications of dexamethasone and aldosterone to fetal hypothalamic cell cultures have been found to inhibit the release of CRH, AVP, and oxytocin through mechanisms involving the activation of protein kinase A (PKA) and protein kinase C (PKC) [17]. In addition, the administration of cortisol to the medium bathing of the hypothalamic neurons of fetal sheep significantly inhibited the potassium-induced secretion of AVP [18]. There is also evidence that the secretion of several steroid hormones is sex-dependent [19,20].

It is possible that during chronic stress glucocorticoids and mineralocorticoids may act oppositely on AVP’s release. Hence, the expressions of CRH hnRNA and AVP hnRNA in the parvocellular neurons of PVN are significantly elevated in rats exposed to forced-swim stress and their increase is abolished by the administration of dexamethasone [21]. On the other hand, the application of eplerenone, which is a mineralocorticoid receptor antagonist, alleviated anxiety-like behavior and reduced vasopressin and corticosterone concentrations in the posterior pituitary [22].

Thus far, multiple gaps exist in understanding the mutual interactions between vasopressin and steroid hormones in health and cardiovascular diseases. Therefore, the principal aim of the present review is to analyze the cooperation of vasopressin and steroid hormones at the cellular and systematic levels in the context of their influence on cardiovascular regulation, tissue metabolism, and oxygenation. Specifically, we discuss the positive and negative consequences of the interaction of AVP and steroid hormones in the development of hypertension and heart failure.

## 2. Interactions of the Hypothalamo–Pituitary–Adrenal System with Vasopressin at the Cellular Level

### 2.1. Genomic and Nongenomic Actions of Steroid Hormones

Multiple studies show that steroid hormones exert both rapid and delayed cellular effects that are mediated by specific types of non-genomic and genomic receptors located either in the cellular membrane or intracellularly [23,24,25,26]. The receptors of specific steroids are encoded by different genes and their activation may result either in stimulatory or in inhibitory effects, depending on the cell type. 

In the individual cell, steroid ligands cooperate with local transcription factors and other regulatory compounds [27,28]. The essential role of binding to receptors and mobilizing posttranslational modifications is played coactivators, which are involved in the integration of cellular processes and the adjustment of cellular responses to current needs. An especially important role is attributed to steroid receptor coactivators (SRCs, namely SRC1, SRC2, and SRC3), which are known as Nuclear Receptor Coactivators (NCOASs), and to Coactivator Binding Inhibitors (CBIs) [29,30].

Mineralocorticoid receptors (MRs, MCRs), glucocorticoid receptors (GRs), estrogen receptors (ERs, ESRs), androgen receptors, and progesterone receptors (PGRs) belong to a subfamily three of nuclear receptors possessing the ligand binding domain (LBD), the DNA-binding domain (DBD), and the N-terminal domain (NTD). In their inactive state, the receptors are present in the cytoplasm in a multiprotein chaperon complex, which contains the ligand-binding cleft identifying and binding the ligand. Binding elicits conformational transformations within the complex that permit the subcellular trafficking of the ligand to the target within the cell and its interaction with DNA [31,32].

*Glucocorticoid and Mineralocorticoid Receptors.* In humans, the function of glucocorticoids is served by cortisol, whereas in the rat it is served by corticosterone, which acts also as a mineralocorticoid. Glucocorticoid and mineralocorticoid receptors are members of the nuclear receptor superfamily of transcription factors (TFs) that modulate processes of transcription through direct binding to the glucocorticoid response element (GRE) or mineralocorticoid response element (MRE) in DNA. A DNA-binding domain is 96% identical in GRs and MRs. The receptors possess also a C-terminal ligand-binding domain (CT-LBD) and an amino-terminus domain (NTD). GR is a 97 kDA protein encoded by the *NR3C1/Nr3c1* gene (in humans located in chromosome 5) and cooperates with several co-regulators [32,33,34]. An amino-terminus contains AF-1 and AF-2 regions which interact with CT-LBD and can stimulate transcription in the absence of a ligand [35,36,37,38,39].

The binding of the ligand by a GR initiates a cascade of events enabling the translocation of the ligand-bound receptor to the nucleus. In the nucleus, the receptors bind to specific DNA sequences which are known as glucocorticoid response elements (GREs) and negative glucocorticoid response elements (nGREs) [40,41]. The regulation of GREs appears to play a dominant role in the cellular processes of neurons [23,40]. The direct occupancy of nGRE results in the repression of the target gene. Steroid receptor coactivators (SRCs) appear to participate in the repression of CRH expression by GRs in the hypothalamus. In the nucleus, MRs and GRs can also interact with some other active proteins (MAZ—myc-associated zinc finger protein, AP-1—activator protein 1, NF-κB—nuclear factor κB, and SRC-1/2/3) that operate as ligand-selective co-regulators. They can induce a remodeling of gene conformation and may initiate the formation of transcription-initiation complexes. GRE-DNA interactions are modulated by chromatin configurations. In the cardiovascular system, the expression of MRs is higher in males than in females [42].

Glucocorticoid receptors have also been identified in the mitochondria, where they regulate mitochondrial gene transcription. In neuronal mitochondria, GRs interact with Bcl-2 protein and form GR/Bcl-2 complexes. Interestingly, a short action exerted at a low density intensifies the formation of complexes, whereas, in high doses, cortisol exerts opposing effects [43]. The interaction of Bcl-2 with other regulatory factors determines the specificity of the actions of glucocorticoids in various organs [37,44,45].

Glucocorticoids also modulate the process of transcription indirectly by physical interaction (tethering), which does not require direct contact with DNA but engages the activation of transcription factors. In various types of cells, these factors may act either as co-activators or as co-repressors. The tissue–cell-dependent expression of co-regulators causes specific the tissue–cell action of steroid molecules [30,37,46,47,48]. It is likely that protein–protein interactions mediate the rapid effects of steroid hormones and play an essential role in the trans-repression of genes by glucocorticoids in hypoxia and inflammatory processes. For instance, it has been found that they interact with hypoxia-induced factors (HIFs) at the level of the promoter region of the inflammatory genes and can either enhance or inhibit the activation of the HIF pathway [47,49]. It has been postulated that, during inflammatory processes, the co-activating function of steroids determines collagen synthesis, the generation of reactive oxygen species, and the engagement of peroxisome proliferator-receptor gamma co-activator 1-alpha (PGC-1α), as well as the activation of p38 mitogen-activated protein kinase and nicotinamide adenine dinucleotide phosphate oxidases (NOX) 2 and 4 [50,51]. On the other hand, glucocorticoids can inhibit inflammation through the repression of genes engaged in the synthesis of pro-inflammatory proteins (AP-1, NFκB) and through the enhancement of the expression of genes involved in the generation of anti-inflammatory compounds [47,52].

There is evidence of reciprocal interactions between glucocorticoid receptors’ pathways. For instance, it has been shown that GRs can induce the expression of genes that promote or inhibit the p38 MAP kinase pathway (MAPK) [51]. Furthermore, the expression of GRs and their responsiveness to glucocorticoids is regulated by microRNAs, whereas the expression of microRNA is regulated by glucocorticoids [53].

The mineralocorticoid receptor is also known as the nuclear receptor of subfamily 3, group C, member 2 (NR3C2). MRs bind mainly aldosterone, but they also show high affinity to cortisol and androgens. The importance of cortisol and aldosterone in specific cell types largely depends on the availability of 11-β-hydroxysteroid dehydrogenase type 2 (11β-HSD2), which converts active cortisol into inactive cortisone. The opposite action is exerted by 11β-HSD1, which transforms cortisone into cortisol. The availability of 11β-HSD2 in several regions of the brain causes aldosterone to have good access to brain MRs and be able exert potent regulatory effects in spite of the fact that its concentration in plasma is hundreds of times lower than the concentration of cortisol [54,55,56]. In the heart, cardiomyocytes and macrophages do not express 11β-HSD2 and both cortisol and aldosterone participate in MR stimulation. Moreover, in the heart, aldosterone exerts some effects through cross-talk with cardiac G-protein-coupled receptors (GPCRs) [57]. Activated MRs can form homodimers or can associate with GRs and form heterodimers.

MRs are present in the kidney, heart, and vessels, where mineralocorticoids participate in the regulation of hypertrophy, fibrosis, inflammation, and apoptosis. Mineralocorticoids can act either directly on NR3C2 receptors or their action can be mediated by the formation of other active molecules, such as interleukin-1 (IL-1), tumor necrosis factor α (TNF-α), cardiotrophin-1 (CT-1), and Toll-like receptor 4 (TLR-4). During the inflammatory process, inflammatory cytokines (IL-1, IL-6, TNF-α) act synergistically with mineralocorticoids and can act jointly through the inhibition of ACTH secretion in the hypothalamic–pituitary–adrenal axis [58]. In the rat’s mesangial cells, aldosterone was found to stimulate NF-κB and glucocorticoid-inducible protein kinase-1 (SGK1) activities. It also elevates promoter activities and the protein expression of intercellular adhesion molecule–1 (ICAM-1) and connective tissue growth factor (CTGF). There is evidence that these factors are involved in aldosterone-mediated mesangial fibrosis and inflammation [59].

In cardiomyocytes, the genomic action of aldosterone mediated by MRs participates in the regulation of chronotropic and hypertrophic actions. It has been shown that aldosterone enhances the expression of mRNA which codes for the α1H protein, and the latter is a constituent of CaV3.2 channel, which is one of the two T channels of cardiomyocytes [60,61,62]. The action of aldosterone on the T channels is presumably indirect, because the gene *CACNA1h*, which codes for the CaV3.2 T-type channel, does not possess an MRE. The increased formation of reactive oxygen species (ROS) and their involvement in the regulation of the affinity of steroid hormones to MRs should also be taken into consideration [63,64,65,66]. In the cardiovascular system, the genomic and non-genomic effects of aldosterone are also modified by angiotensin II (Ang II) [39].

In the brain, MRs have been identified mainly in the hippocampus, septum, and other limbic structures, whereas GRs are expressed in the septum, hippocampus, brain stem, and the prefrontal cortex [44,65,66,67]. The affinity of corticosterone to the MRs in neurons is 10-fold higher than to their GRs [65,66,67]. MRs are associated with the cellular membrane and, after their activation, they are translocated with the help of β-arrestin to the cells where they can exert their action through non-genomic GPCR processes [25,57].

*Androgen, Estrogen, and Progesterone.* Both in males and females, androgens are synthesized in the adrenal glands (mainly in the *zona fascicularis* and the *zona reticularis*), in the brain (mainly in the hippocampus), and in the liver [68]. In males, testosterone is produced chiefly by the Leydig cells of the testes, while, in females, testosterone and its metabolites are produced primarily in the adrenal glands and ovaries. The cells of the adrenal cortex also synthesize dehydroepiandrosterone (DHEA), androstenedione, androstendione, androstenediol, and 11-β-hydroxyandrostenedione. Testosterone can be converted to dihydrotestosterone by 5α-reductase, while deoxycorticosterone is converted into dihydrodeoxycorticosterone. Both DHEA and testosterone are able to stimulate androgen receptors; however, DHEA has significantly greater androgenic activity than testosterone. Aromatase-producing dihydrotestosterone is also involved in the formation of estradiol, which is engaged in the stimulation of estrogen receptors. This gene, which encodes aromatase (*CYP19A1*), is located on chromatosome 15 and has been identified in the lungs, vessels, and multiple brain regions [69,70]. The process of the aromatization of testosterone to estradiol occurs in several peripheral tissues and in the brain [68].

Androgens, estrogens, and progesterone interact with receptors in the brain, heart, and vessels and participate in the regulation of the cardiovascular system by means of classic genomic and non-classic pathways [71,72,73,74,75,76,77,78]. Androgen receptors were identified in cells of the reproductive system, bones, vessels, and brain [78,79]. In the brain, ARs are present in the cortex, midbrain, brain stem, and spinal cord. Specifically, a high density of AR immunoreactivity was found in the olfactory bulb, the nucleus accumbens, the medial amygdala, the bed nucleus of the stria terminalis, the medial preoptic area, the septum, the mesencephalic periaqueductal gray (PAG), the dorsal raphe nucleus, the substantia nigra, the area postrema, the dorsal motor vagus nucleus, and in the preganglionic cells of the autonomic nervous system [75,77].

Androgen signaling engages several molecular pathways. The primary androgen receptor is a nuclear transcription factor that is activated mainly by testosterone and dihydrotestosterone. Non-stimulated ARs are present mainly in the cytoplasm and are associated with heat shock proteins (HSPs). The association of androgen with ARs allows the dissociation of these receptors from chaperone proteins and the translocation of the androgen–AR complex to the nucleus, where it binds to the androgen-response element (ARE) and regulates gene transcription. Androgens also regulate rapid non-genomic processes, engaging G-protein-coupled receptor family C (GPRC6A), zinc transporter ZIP9 membrane-receptor, and oxoeicosanoid receptor (OXER). Consequentially, through their activation of the genomic-dependent and non-genomic dependent signaling pathways, androgens initiate transcription processes and activate canonical pathways associated with the activation of ionotropic receptors, G-protein-coupled receptors activating PLC, calcium transporters, and endothelial nitric oxide synthase (eNOS). Testosterone can also stimulate membrane ARs, which bind to Src and activate the MAPK pathway. The transactivation of membrane ARs by other ligands has also been reported [78,79]. Most likely, the activation of rapid non-genomic processes is essential for the fast action of androgens, such as their cell migration, mitosis, and inflammatory processes [80,81,82].

Estrogens easily penetrate the cellular membrane, reaching their highest concentration within the nucleus compartment [83,84,85]. In cells, they regulate long-lasting processes by means of nuclear receptors and rapid non-genomic processes. Nuclear estrogen receptors ESR1 (ERα) and ESR2 (ERβ) are codified by the *ESR1* and *ESR2* genes. *ESR1* is located on chromosome 6 (6q25.1) and *ESR2* on chromosome 14 (14q23.2). Both receptors act as transcription factors that mediate the transcriptional activity of estrogens with reference to specific genes. In the absence of the ligand, ESRs are associated with HSP and do not express transcriptional activity. After activation by the ligand, ESRs interact with the estrogen response element (ERE) and operate either as monomers or as dimers (ESR1-ESR1; ESR2-ESR2 or ESR1-ESR2). In the nucleus, estrogens enhance the transcription of specific target genes [74,83,84,85] Estrogens can also modulate the expression of other genes acting indirectly via the activation of PI3K/Akt and MAPK/ERK pathways, as well as through the inhibition of the JNK pathway [86].

Multiple essential actions of estrogens, such as the inhibition of ROS production, the regulation of mitochondrial ATP levels, and the formation of mitochondrial structural conglomerations, occur in the mitochondria [87,88]. Recently, attention has been drawn to the essential role of estrogens in the regulation of mitochondrial bioenergetics in human subjects [89]. Estrogens also interact with the membrane-associated G-protein-coupled receptor (GPER, named GPR30) which is present in the endoplasmic nucleus, Golgi apparatus, and cellular membrane. GPERs are involved in the rapid non-genomic actions of estrogens that are mediated by extracellularly activated kinase (ERK), cyclic adenosine monophosphate (cAMP), and Ca^2^. With regard to the cardiovascular system, it is essential to note that the stimulation of estrogen receptors results in the activation of several rapid and long-lasting cellular processes which are essential for the function of the heart and vessels. Functionally effective ESRs are present in the cardiac and vascular smooth muscle cells and modulate the function of the perivascular unit [90,91]. GPERs, ERα, Erβ, and GPRE1 are widely represented in most cell types of the cardiovascular system and in the adipose tissue [86,92,93]. The processes of the stimulation of ERs involve the activation of the phosphoinoisitide 3-kinase-serin/threonine-specific kinase B (PI3K/Akt/eNOS) and mitogen-activated protein kinase MAPK/eNOS pathways, which are engaged in the production of NO and play an essential role in vasodilation. Moreover, it has been shown that, in cardiomyocytes, estrogens regulate the activity of calcium-handling proteins, including L-type Ca^2+^ channel (LTCC), ryanodine channel (RYR), sarcoplasmic reticulum Ca^2+^ ATPase, and sodium–calcium exchanger (NCX). Thus, it has been suggested that complex reciprocal interactions between the activation of estrogen receptors and calcium signaling pathways may play an essential role in the regulation of cardiomyocytes’ activities [73,94]. Furthermore, it has been reported that estrogens exert an antioxidant action and regulate cell contractility through effects exerted on the calcium-dependent signaling pathways operating in the cardiac mitochondria and sarcoplasmic/endoplasmic reticulum, where they modulate Ca^2+^-ATPase 2a (SERCA2a) activity and the function of calcium ion channels [73,95,96,97,98]. Altogether, it is likely that, in the heart, a deficiency of estrogen may result in disturbances in calcium homeostasis. It has also been reported that the interaction of ERα with peroxisome proliferator-activator receptors (PPARs) causes a repression of the transactivation of the PPAR in the heart and vessels [86].

It is likely that estrogens may also influence the function of the cardiovascular system through actions exerted in the brain as their receptors are widely expressed in multiple brain regions involved in cardiovascular regulation, such as the frontal cortex, the sensorimotor cortex, the thalamus, the hypothalamus, the amygdala, the ventral tegmental area, the hippocampus, the dorsal raphe nucleus, and the cerebellum [72,99]. Finally, it should be noted that the activation of ERα and GPER1 plays a significant role in the modulation of the immune processes that are activated in cardiovascular diseases [74,84,91,100,101].

The action of progesterone (P4) is mediated through genomic signaling, engaging two subtypes of nuclear receptors (PGRA, PGRB), and through non-genomic G-protein-associated membrane-progestin receptors (mPRs). Some actions of PG can also be exerted by GRs [102,103,104].

*Neurosteroids.* Some steroids, which are known as neurosteroids, have been identified in the nervous system and act preferably on neuronal membrane receptors. Among them are steroid sulfates, such as DHEAS, which is a product of the sulfation of DHEA [105]. It is suggested that neurosteroids play a significant role in the modulation of the action of other steroids and classical neurotransmitters. Their action is not a subject of discussion in the present survey (see [103,104,105,106] for a further review of this topic).

### 2.2. Genomic and Non-Genomic Effects of Vasopressin

Vasopressin, which is a principal vasopressin peptide in mammals, is synthesized mainly in the neurons of the hypothalamic supraoptic, paraventricular, and suprachiasmatic nuclei. The majority of the axons of these neurons reach the posterior pituitary, where AVP is released into the blood and can be distributed to peripheral organs. Some of the axons reach the median eminence and release AVP into the hypophyseal portal system and the anterior pituitary, where vasopressin contributes to the regulation of ACTH. Vasopressin-expressing cells have also been identified in the brain regions engaged in the regulation of blood pressure, metabolism, pain, stress, and anxiety. Among them are neurons of the brain cortex, olfactory bulb, BNST, dorsomedial hypothalamic nucleus, nucleus of the diagonal band of Broca, circumventricular organs, brain stem, and spinal cord [107,108,109]. In addition, AVP mRNA has been detected in the heart and vessels and in pancreatic tissue [110,111].

The vasopressin gene is located in chromosome 20 and consists of three exons (A, B, and C). The exon A codes for a signal peptide, vasopressin peptide (Cys-Tyr-phe-Gln-Asn-Cys-Pro-Arg-Gly-NH2), a three-amino acid spacer, and the first nine (NH_2_ terminal) aminoacids of neurophysin (NP). Exon B codes for the mid-portion of NP, whereas exon C codes for the terminal portion of NP, a cleavage site, and the COOH-terminal glycopeptide (GP, known as copeptin). Copeptin consists of 39 aminoacids and is released from neurons in equimolar quantities with AVP [112,113]. Because the copeptin molecule is more stable than the vasopressin molecule, measurements of GP concentrations are frequently used as a marker of AVP levels. Measurements of GP levels have been included in ESC guidelines on myocardial infarction and as a biomarker of inflammation [114,115,116].

The expression of the vasopressin gene is influenced by changes in body fluid’s osmolality and blood volume, as well as by constituents of the hypothalamo–hypophysial–adrenal axis, pain, cytokines, and inflammation factors, especially those associated with COVID-19 [113,117,118,119,120,121]. Vasopressin gene expression and AVP mRNA abundance are enhanced by chronic osmotic simulation and are decreased by hypoosmolality. The osmotically induced increase in AVP mRNA and the release of AVP by neurons of the hypothalamo–neurohypophyseal axis are potentiated by the administration of lipopolysaccharide and the enhanced release of IL-1β and IL-6 in the posterior pituitary [122]. The expression of the AVP gene in the hypothalamus is potentiated by IL-1 and IL-2. Moreover, IL-1β stimulates the release of CRH, AVP, and melanocortin-stimulating hormone (α-MSH) [123,124].

It has been shown that hypoosmolality induces GRs’ expression and that this is related to corticosterone-negative feedback on AVP transcriptions. The above data support the hypothesis that the AVP gene is directly inhibited by glucocorticoids and that the induction of GRs in the hypothalamic cells suppresses AVP expression during prolonged hypoosmolality [112]. However, it should be noted that during prolonged increases in corticosteroids’ concentrations, such as the one takes place during autoimmune inflammation, vasopressin neurons can escape from glucocorticoids’ inhibition, presumably due to the increased engagement of inflammatory cytokines [125].

Osmotic stimulation induces the rapid upregulation of CRH in vasopressinergic neurons of the hypothalamic magnocellular nuclei [126]. There is evidence of the coordinated regulation of AVP and CRH genes by glucocorticoids. It has been shown that adrenalectomy causes the enhancement of CRH and AVP immunoreactivity in the hypothalamus and elevated CRH immunoreactivity in the cerebral cortex, the amygdala, and the BNST. Since the stimulatory effect of adrenalectomy on the expression of AVP and CRH in the hypothalamus could be reduced by the administration of dexamethasone, it was concluded that glucocorticoids produced in the adrenal glands play a primarily inhibitory role in the regulation of AVP and CRH secretions [127]. The PVN neurons express glucocorticoid receptors and glucocorticoids reduce AVP gene expression in the parvocellular neurosecretory neurons of the PVN [128]. The AVP gene and CRH gene possess cAMP response elements (CREs), that are activated by intracellular cAMP, and AP1 and AP2 transcription factors can be repressed by glucocorticoids. In the PVN neurons, the expression of the CRH and AVP genes is regulated by nGREs and serum response elements [113,129,130]. Vasopressin neurons also express MRs and there is evidence that aldosterone and corticosterone increase the sodium channel (ENaC) leak current through an action exerted at the promoter region of the γ ENaC gene [131]. The magnocellular neurosecretory neurons of the PVN and SON, as well as of other brain regions, express 11β-HSD2, and this increases their sensitivity for aldosterone [56,132].

It appears that, during restraint stress, the inhibitory effect of glucocorticoids on CRH and AVP mRNA expressions in the PVN neurons can be modulated by the concomitant release of testosterone [133]. During chronic stress, glutamergic, gamma-aminobutyric acid (GABA), and noradrenergic terminals exert a number of convergent actions that jointly regulate the activity of the CRF and AVP neurons of the PVN [134]. Growing evidence indicates that androgens play an essential role in the regulation of neurons expressing CRH, AVP, dopamine, and serotonin during stress-related behavior [135].

*Vasopressin receptors.* Arginine vasopressin stimulates two subtypes of V1 receptors (V1R), known as V1aR and V1bR, and one type of V2 receptor (V2R). The AVP receptors belong to a family of G-protein-coupled receptors. In high concentrations, AVP also interacts with oxytocin receptors [109,136,137,138,139]. The AVPR1a gene has been mapped to the 12q14.2 locus and AVPR1b to the 1q32.1 locus. The AVP2R gene is located on the long arm of the X-chromosome (Xq28). The mutation of the V2R gene is inherited in an X-linked manner and results in congenital nephrogenic diabetes insipidus, which is characterized by strong polydipsia and polyuria [140,141]. It has been shown that vasopressin receptors can act as homodimers and as heterodimers and it is likely that their dimerization influences the effectiveness of the stimulation of the target cells. Specifically, the formation of V1aR and OTR and V1bR and corticotropin-releasing hormone receptor (CRHR) heterodimers has been well documented [142,143]. The expression of vasopressin receptors is regulated by corticosteroids [144].

Vasopressin V1aR mRNA and protein have been detected in multiple organs and tissues, including the heart and vessels, and their expression is altered in pathological processes [113,138,139,140,141,142,143,144,145]. In cardiac ventricular sarcolemma, vasopressin was found to open K_ATP_ channels through an action exerted on V1R [146]. AVP was also found to reduce Ca^2+^ influx through L-type Ca^2+^ channels, and this effect can be abolished by a blockade of V1aR [147]. There is evidence that the stimulation of cardiac V1aR decreases cardiac beta receptors’ responsiveness [148].

In vitro experiments on H9c2 rat ventricle cardiomyocytes exposed to hypoxia revealed that AVP acting on V1aR in a V1aR/GRK2/β- arrestin1/ERK1/2-dependent manner enhances cell survival. It has been suggested that, during heart failure, when the levels of circulating AVP are elevated, the inhibition of G protein-coupled receptor kinase 2 (GRK2) can potentially exacerbate negative V1aR-mediated effects by preventing receptor desensitization and augmenting Gαq protein-dependent signaling [149,150]. In the heart, the stimulation of V1aR is also engaged in the generation of pro-inflammatory cytokines and in the development of inflammation and fibrosis. AVP increases IL-6 mRNA and protein levels in cardiac fibroblasts and this effect requires the activation of GRK2 and NF-κB [151]. In addition, it has been shown that endotoxemia induced by lipopolysaccharides and the concomitant increase in IL-1β, TNF-α, and interferon gamma causes a downregulation of V1aR gene expression in the heart, vessels, liver, and lungs, as well as a reduction in the responsiveness of vascular smooth muscle cells. It is likely that the diminished responsiveness of V1aR to vasopressin accounts for its inadequate stimulation during the circulatory shock that can occur during endotoxemia [152].

A growing number of studies provide evidence of the interaction of AVP with corticosteroids in other organs, whose proper action is necessary for the appropriate regulation of cardiovascular functions, such as the brain and the gastrointestinal system. The administration of corticosterone was found to decrease the expression of V1aR in the lateral septum and the hippocampus [144]. The stimulation of V1aR in cortical astrocytes was found to exert a neuroprotective action and this effect was associated with the activation of the nuclear transcription factor cAMP response element-binding protein (CREB) and with a prominent decrease in IL-1β and TNF-α gene expressions [146]. Vasopressin also plays an essential role in the regulation of the gut microbiome [151]. The enhanced stimulation of V1aR may participate in the habituation of the release of ACTH, corticosterone, and testosterone to repeated stress in Sprague Dawley rats [153].

V1b receptor mRNA is expressed in the corticotropic cells of the brain and in the anterior pituitary, the adrenal gland, the heart, the kidney, the thymus, the lung, the spleen, the pancreas, the uterus, and in white adipose tissue [154,155,156]. In the brain, V1bR mRNA was detected in the olfactory bulb, the cortex, the hippocampus, the hypothalamus, the septum, and the cerebellum; however, its expression was lower than the expression of V1aR mRNA [156]. In the pancreas, stimulation of V1bR potentiates the secretion of insulin from β cells where AVP acts synergistically with CRH [156]. Thus far, there is no convincing evidence of the presence of V2R in the heart and vessels and of their direct involvement in the regulation of cardiovascular functions via vasopressin. Nevertheless, through the stimulation of V2R in the kidneys and through the regulation of urine concentrations and body fluid natremia, AVP may modulate the effects of the stimulation of V1R in the cardiovascular system [153,154,155,156,157,158].

## 3. Role of the Hypothalamo–Pituitary–Adrenal System and Vasopressin in the Regulation of Energy Balance and Water–Electrolyte Balance at Rest and during Neurogenic Stress

### 3.1. Regulation of Steroids Secretion

**Figure 2:**
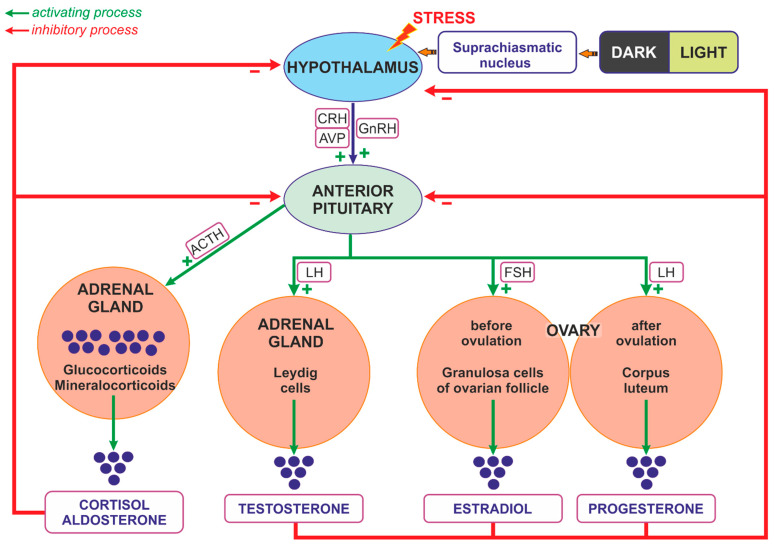
The regulation of the secretion of hormones of the hypothalamo–pituitary–adrenal axis. ACTH—adrenocorticotropic hormone, AVP—arginine vasopressin, CRH—corticotropine-releasing hormone, FSH—follicle-stimulating hormone; GnRH—gonadotropine-releasing hormone, LH—luteinizing hormone, + stimulation, - inhibition. Other explanations are in the text.

The synthesis of glucocorticoids in the adrenal cortex is regulated mainly by ACTH, which is released from the pituitary gland. The secretion of ACTH is closely regulated by CRH and AVP, which are supplied principally by neurons of the PVN [158,159,160]. Glucocorticoids released from the adrenal cortex and ACTH secreted from the corticotropic cells of the pituitary gland are able to inhibit the reciprocal secretion of CRH from the hypothalamic neurons [113,129,161] (Figure 2).

**Figure 3:**
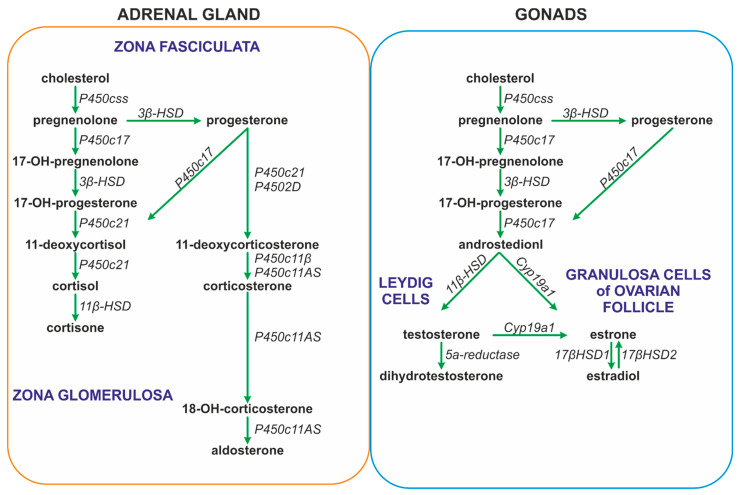
Steps of the synthesis of steroid hormones in the adrenal glands and gonads. Other explanations are in the text.

The secretion of glucocorticoids from the adrenal cortex is regulated by the circadian rhythm; however, the daily rhythm of glucocorticoid release may be influenced by other factors, such as gender, aging, early life stress, inflammatory diseases, pregnancy, and lactation [159,162]. ACTH binds on the surface of cells of the fascicular layer of the adrenal cortex with the G protein-coupled melanocortin type 2 receptor (MC2R), which activates the 3′,5′-cyclic AMP signaling cascade, enabling the penetration of cholesterol into the inner mitochondrial membrane. Cholesterol is converted by cytochrome P450 into pregnenolone, which is converted in several steps to progesterone, 11-deoxycortisol, and cortisol (the main glucocorticoid in humans), or corticosterone (the main glucocorticoid in rodents) (Figure 3) [159].

ACTH also stimulates the synthesis of aldosterone in the glomerular layer of the adrenal cortex. Aldosterone is synthesized from cholesterol in the inner mitochondrial membrane with the participation of aldosterone synthase (CYP11B2). The process of its synthesis includes the formation of pregnenolone and the conversion of pregnenolone to 11-deoxycorticosterone (DOC) in the smooth endoplasmic reticulum (Figure 3). Further processing to aldosterone takes place again in the mitochondria [163,164]. The synthesis of aldosterone in the adrenal cortex can also be stimulated by the renin-angiotensin system (RAS) and by extracellular potassium concentrations [163].

Androgens, as well as estrogen and progesterone, are released during the activation of the HPA, which plays a crucial role in the regulation of the ovarian cycle. The hypothalamic cells synthesize GnRH, which stimulates the release of LH and FSH from the anterior pituitary gland. In the woman, FSH stimulates follicular maturation and the release of estrogen in the follicular phase, while LH triggers ovulation as well as the release of progesterone from the corpus luteum *(ruptured follicle*) in the luteal phase. Estrogens released from the ovaries and acting through reciprocal inhibition are able to slow down the activity of both the pituitary gland (the synthesis and secretion of FSH and LH) and the hypothalamic neurons (the synthesis and secretion of GnRH), thereby generating the menstrual cycle [164,165]. Estrogens are synthesized as a result of the action of the aromatase, which is responsible for the aromatization of androgens into estrogens (Figure 3) [166]. The main site of the expression of aromatase in premenopausal women is located in the ovarian granulosa cells. In pregnant women it is also present in the placental syncytiotrophoblast. Both in men and in postmenopausal women, fibroblasts of adipose tissue and skin contribute to estrogen synthesis [167]. During reproductive life, women show a characteristic periodicity in their secretion of ovarian hormones, which corresponds to the monthly menstrual cycle. 

As already mentioned, androgens are synthesized mainly in adult female ovaries and in male testes. In addition, their synthesis is stimulated by ACTH in the reticular zone of the adrenal glands (mainly dehydroepiandrosterone sulfate, DHEA/-S; 4-androstenedione, A4; and 11β-hydroxyandrostenedione). Androgens are metabolized in the liver and subsequently excreted by the kidneys [168]. In men, the dominant androgen is testosterone; its concentration is 15 times higher than in women [169,170]. Testosterone synthesis in men takes place in the Leydig cells of the testes (Figure 2 and Figure 3). The activation of the Leydig cell LH receptors by LH initiates an intracellular signaling cascade, which involves the stimulation of adenylate cyclase, the increased production of cAMP, and the activation of adenosine-dependent kinase [171]. As a consequence of the activation of cAMP-dependent intracellular signal transduction, cholesterol can be transferred to the mitochondria by the steroidogenic acute regulatory protein (STAR), the translocator protein (18 kDa; TSPO), and other transducosome proteins [172]. In the mitochondria of Leydig cells, cholesterol is converted to pregnenolone by the cytochrome P450 enzyme located on the matrix side of the inner mitochondrial membrane, which cleaves the C27 cholesterol side chain (CYP11A1). Subsequently, after transfer to the smooth endoplasmic reticulum, pregnenolone is metabolized to testosterone by 3β-hydroxysteroid dehydrogenase (3β-HSD; HSD3B), 17α-hydroxylase/17,20 lyase (CYP17A1), and 17β-hydroxysteroid dehydrogenase type 3 (17β-HSD3, HSD17B) (Figure 3) [173,174,175].

### 3.2. Interaction between Steroids and AVP in the Regulation of Energy Balance

Multiple studies indicate that both steroid hormones and vasopressin play an important role in the regulation of energy balance. Glucocorticoids promote glucose production in the liver and reduce peripheral glucose uptake by the muscle and adipose tissue. They also increase the breakdown of the constituents of fat and muscle, thereby providing additional substrates for glucose and free fatty acids’ production [176].

Vasopressin is involved in the regulation of food intake and energy balance via a wide range of central and peripheral actions exerted on cellular growth and proliferation, protein turnover, lipid metabolism, and glucose homeostasis [177].

#### 3.2.1. Animal Studies

It has been shown that endogenous nucleobindin-2 (NUCB2)/nesfatin-1 regulates AVP and oxytocin in the PVN of male mice, and this is associated with a resetting of fluid balance and food intake, and with an increase in body weight without a change in energy expenditure [178]. Experiments on mice showed that the administration of an AVP analog (Ac3IV) twice a day for 22 days not only reduces energy consumption, body weight, and fat content, but also decreases blood glucose concentration, increases insulin sensitivity, and significantly improves glucose tolerance and glucose-induced insulin secretion. Moreover, it lowers total cholesterol and low-density lipoprotein (LDL) cholesterol and triglycerides and increases high-density lipoprotein (HDL) cholesterol [179]. Several hormones affecting energy balance play a significant role in the regulation of AVP secretion. Ghrelin, an orexigenic hormone, has been shown to stimulate vasopressinergic neurons in the hypothalamic paraventricular nucleus and AVP secretion in a nutritional-status-dependent manner in in vivo experiments. It also activated excitatory GABAergic synaptic input via a retrograde neuron–glial circuit in hypothalamic slices obtained from fasted rats [180]. On the other hand, the cerebroventricular administration of adiponectin significantly reduced basal plasma AVP concentrations in conscious rats in a dose-dependent manner, with the maximum effect achieved 10 min after administration [181].

Additionally, it appears that AVP may exert thermogenic functions, as the exposure of brown adipose tissue (BAT) adipocytes to AVP was found to increase uncoupling protein-1 (UCP-1) protein expression, induce a time- and dose-dependent increase in p38 MAP kinase phosphorylation, and increase monocyte chemoattractant protein-1 (MCP-1) mRNA and IL-6 expressions. In contrast, adiponectin mRNA expression was reduced [182]. Former studies have shown that acting, in the adipose tissue, on V1aR AVP may exert both lipolytic and antilipolytic effects [183,184]. Its action on lipid metabolism through V1bR is different. It has been shown that V1bR-deficient mice have a lower body weight and greater epididymal adipose tissue mass in comparison to wild-type mice. It is believed that AVP acting on V1bR receptors is able to influence lipid metabolism through the modulation of insulin signaling. Currently, it appears that the stimulation of V1aRs results in the impairment of glucose tolerance and in lipolytic action, whereas the activation of V1bRs improves glucose tolerance and exerts an antilipolytic effect [185,186].

#### 3.2.2. Human Studies 

Elevated plasma copeptin (AVP substitute) concentrations have been demonstrated in patients with type 2 diabetes and with type 1 diabetes [187]. Moreover, recent studies indicate that higher concentrations of AVP or copeptin predispose patients to the development of type 2 diabetes and metabolic syndrome and that the consumption of larger amounts of water by people with high plasma copeptin concentrations results in a reduction in fasting glucose or glucagon levels [188]. Studies on human populations revealed an association of polymorphisms of the gene encoding AVP with metabolic disorders. A significant relationship was found between high copeptin levels and reduced insulin sensitivity, as well as between AVP gene tagSNPs (CC genotype rs6084264, TT genotype rs2282018, C allele rs2770381, and CC genotype rs1410713) and the incidence of hyperglycemia [189]. Similarly, a specific polymorphism of the V1aR gene (T allele of rs1042615) has been associated with an increased incidence of type 2 diabetes in people who consume large amounts of fat or are overweight [190]. In another study, the major A allele of rs35810727, a tagSNP of the V1bR, has been associated with an increased body mass index (BMI) and type 2 diabetes [191].

### 3.3. Interaction of AVP and Steroids in the Regulation of Water–Electrolyte Balance

It is well known that the vasopressin system (VS) plays a pivotal role in the regulation of water and electrolytes’ balance and that a lack of AVP results in the excretion of large amounts of free water in the urine (polyuria) and in subsequent polydipsia [108,192,193,194]. The release of AVP is regulated by a variety of internal and external cooperating factors, acting as anticipatory or consecutive signals [192,194,195,196,197,198,199]. The regulation of water–electrolyte balance via AVP is a complex process in which the osmolality of the extracellular fluid (ECF), including the plasma and cerebrospinal fluid, plays a key role. ECF tonicity is sensed by osmoreceptive neurons located mainly in the subfornical organ (SFO) and the organum vasculosum of the lamina terminalis (OVLT), which are able to sense osmolarity using the aquaporin (AQP) receptor and have direct connections with the PVN and SON [200,201]. The osmotically induced shrinkage of osmosensitive cells during dehydration causes the activation of TRPV1 delta-N channels (a family of transient receptor potential cation channels) that sense mechanical stretch and allow the influx of cations into the cell [200,202]. In addition, the brain osmoreceptors’ activity may be regulated by anticipatory signals generated in receptors in the oropharyngeal region, esophagus, or stomach, which participate in the regulation of fluid intake. Specifically, information about the taste of water is transmitted centripetally by the tympanic cord belonging to the facial nerve, information about dry mouth is received via the trigeminal nerve, and information about the stretching of the esophagus and stomach and the volume of water ingested are transmitted by the glossopharyngeal nerve and by the vagus nerve [203].

In the kidneys, AVP regulates the activity of the aquaporin-2 (AQ2) water channel in the collecting duct and participates in the process of urine concentration [204]. Moreover, it stimulates sodium reabsorption, acting on the luminal sodium channel ENaC in the cortical and outer medullary parts of the collecting tubules, and activates urea transporters UT-A1 and UT-A3 in the inner terminal medullary, increasing urea reabsorption in the collecting tubules [205,206]. Additionally, at higher concentrations, AVP also increases sodium reabsorption through the activation of Na-K-2Cl cotransporters (NKCC2) in the thick ascending limb of the nephron loop (Henle’s loop) [207].

There is strong evidence that the regulation of water–electrolyte balance by vasopressin is significantly influenced by steroid hormones.

#### Studies on Animals and Human Subjects

Experimental studies have shown that glucocorticoids’ deficiency facilitates the activation of V2 receptors in the renal collecting tubule [208]. It has also been shown that the application of dexamethasone improves cardiac functions in rats with congestive heart failure elicited by coronary artery ligation and that this effect is associated with a reduced expression of V2R and AQP2 and AQP3 and with a reduced number of ENaC and Na-K-2Cl cotransporters 3 in the renal collecting tubule [205]. In rats, the effects of dexamethasone were abolished by the use of the glucocorticoid receptor inhibitor RU486 [205]. However, clinical studies could not confirm the significant involvement of glucocorticoids applied alone in fluid retention in critically ill patients. Therefore, it has been postulated that the effects of steroid hormones on water–electrolyte balance are mediated mainly by the activation of mineralocorticoid receptors [206].

It is well known that mineralocorticoids have a significant impact on electrolyte balance, especially on sodium and potassium turnover, as well as on the clearance of free water [207,208]. 

Currently, it also appears that gonadal steroids have a significant effect on the vasopressinergic system [209]. Experimental studies have revealed that the antidiuretic function of AVP is more effective in male rats than in female rats [209]. Furthermore, its effect is more potent in females during estrus, when the level of circulating estrogen is low [210]. In addition, ovariectomy was found to increase their antidiuretic response to AVP to a level comparable to that observed in males, whereas estradiol substitution reduced the antidiuretic effect of AVP to the level observed in non-estrus females [210]. It appears that estrogens may also influence the hypothalamic vasopressinergic neurons, acting both through the ERα and ERβ receptors. In this context, the presence of ERα on osmoreceptive neurons of the SFO and OVLT, which send projections to the SON, has been demonstrated [211,212,213]. Moreover, both ERα and ERβ have been detected in the kidney, where the expression of ERα is prevailing [214]. Interestingly, GPER is a newly discovered aldosterone receptor that mediates nongenomic aldosterone pathways. GPER activation by aldosterone mediates water and sodium retention in the body and contributes to vasoconstriction [215]. Recently, it has been shown that the production of aldosterone may be influenced by progesterone [216].

It should be noted that testosterone, in addition to estrogen, can directly inhibit AVP secretion [217]. However, it is frequently emphasized that the effect of testosterone, and the action of some of its metabolites (α-androstane-3β), on the release of AVP may be mediated by ERβ [214].

### 3.4. Interaction of AVP and Steroids in Neurogenic Stress

A number of studies provide evidence that neurogenically mediated stress causes s joint stimulation of vasopressin-secreting neurons and CRH-secreting neurons, which suggests that a coordinated regulation of AVP and CRH’s release and action may play an important role in the modulation of neurogenic stress. Moreover, it has been found that, in chronic stress and depression, the effects of CRH on ACTH release are strongly enhanced by vasopressin, which is produced in increasing amounts when the hypothalamic PVN and SON neurons are chronically activated [218,219]. It has been found that AVP is engaged in stress-induced tachycardia and baroreceptor reflex (BRR) desensitization [220]. Clinical studies have shown that healthy adults exposed to a social stress test respond with an increase in serum copeptin concentration [218]. Experiments on mice showed that chronic unpredictable stress (UCS) and ovariectomy influence AVP immunoreactivity in the lateral magnocellular and the medial magnocellular subdivisions of the PVN [221]. The role of vasopressin in the response to stress appears to differ in female and male C57BL/6 mice. Namely, a six-week chronic variable stress (CVS) paradigm has been found to increase sociability in female mice and to decrease AVP mRNA in the PVN, whereas, in male mice, CVS had no effect on social behavior or AVP expression [222,223]. Studies conducted on male Sprague Dawley rats subjected to 4 weeks of unpredictable chronic mild stress (UCMS) showed that injections of small interfering RNA (siRNA) directed against V1aR into the PVN prevent an increase in blood pressure and the elevation of renal sympathetic nerve activity (RSNA), whereas the administration of scrambled RNA (scrRNA) into the PVN elicits an increase in blood pressure. The results suggested that V1aR signaling in the PVN contributes to the generation of neuro-cardiovascular responses to stress [224]. Research performed by Komnenov et al. (2021) showed that rats subjected to UCMS have higher plasma AVP levels and a greater abundance of V1aR and V1bR transcripts in their PVN. The rats also manifested a higher resting MAPK, heart rate, and RSNA, and these effects could be eliminated by the combined inhibition of V1aR and V1bR [225].

More recently, human studies have shown that, during chronic stress, the hypothalamic activation of pituitary changes from the dominant CRH phenotype to the dominant AVP phenotype; however, the cortisol level remains elevated because its metabolism is reduced [222]. In addition, recent studies have provided evidence that the rs10877969 polymorphism of the V1aR gene is associated with the elevation of symptoms of stress and acute pain [226].

#### Sex Differences

It is well known that there are sex differences in the tolerance of stress and in the predisposition to anxiety and depression that may be partly associated with different regulations of the HPA in males and females [227,228]. Experimental studies have demonstrated that female rats respond with larger increases in ACTH to neurogenic stress than male rats and suggested that, during neurogenic stress, the gonadal steroids involved in the regulation of sexual behavior and reproduction may have a potential impact on the pituitary secretion of ACTH [229,230]. Subsequently, it has been shown that the secretion of ACTH is regulated by a testosterone-dependent effect on the synthesis of AVP and by a corticosterone-dependent effect on the synthesis of CRH in the PVN [231]. It is likely that one of the reasons for differences in sex-related susceptibility to stress is the functional heterogeneity of GRs and MRs in the brains of females and males, because a higher expression of MRs was found in the brain of the male mice than in the brain of female mice manifesting depressive behavior [232]. Research performed by Woodward et al. (2023) on female and male mice subjected to the UCMS procedure showed that female mice show anxiety and depressive behavior associated with FosB activation in the neurons of their medial prefrontal cortex (mPFC), expressing parvalbumin after 4 weeks of exposure, while in male mice behavioral and biochemical alterations are observed not earlier than after 8 weeks of UCMS. Furthermore, the use of patch-clamp electrophysiology allowed the researchers to demonstrate that there were time-corresponding sex-specific differences in the altered neuronal excitability of mPFC cells after 4 and 8 weeks of exposure [228]. Rosinger et al. (2019), using a corticotropin-releasing factor 1 receptor (CRFR1) reporter mouse line, demonstrated that male mice showed a significantly higher distribution of cells expressing CRFR1 in their PVN compared to females; however, it should be noted that this relationship was age-related, because it was observed only in older (20–24-month) mice and not in mice during early post-natal life. Gonadectomy in adult six-week-old mice resulted in a significant decrease in the number of CRFR1-immunoreactive cells in the PVN in males but not in females. In addition, their CRFR1 cells showed moderate co-expression with estrogen receptor alpha and high co-expression with androgen receptors. The use of restraint stress resulted in a greater activation of CRFR1 cells in the PVN of male mice than in the PVN of female mice [232]. Cox et al. (2015) examined the association of X chromosome genes with behavioral disorders in an experimental model (fragile X syndrome, autism) using a mouse model with an atypical sex chromosome configuration resembling Turner (45, XO) and Klinefelter (47, XXY) syndromes. The researchers showed higher AVP expression in the amygdala of female mice with one copy of the X-chromosome gene. A reduced level of plasma AVP was found in girls with Turner syndrome [233].

The recent clinical studies of Cohen et al. (2023) have revealed that gender differences in the reaction to stress already occur in young people aged 18–25, and they may be related to the different activation of their prefrontal cortexes. Using GABA in magnetic resonance spectroscopy, the authors were able to demonstrate significant differences in neuronal activity in the responses to stress among the sexes. It should be noted that the differences were especially evident in the ventromedial prefrontal cortex, which plays an essential role in the regulation of the hypothalamic–pituitary, hypothalamic–pituitary–adrenal, and hypothalamic–pituitary–gonadal axes [234].

## 4. Altered Interactions of Vasopressin with the Hypothalamo–Pituitary–Adrenal System in Cardiovascular and Metabolic Diseases

### 4.1. Cardiovascular Diseases

Substantial evidence indicates that the central and peripheral interactions of steroid hormones and vasopressin are reprogrammed in hypertension and that these modifications may initiate and/or potentiate cardiovascular complications. Pietranera et al. (2004) revealed that subcutaneous injections of deoxycorticosterone acetate cause significantly greater increases in AVP and V1aR mRNA in the magnocellular divisions of the PVN in spontaneously hypertensive (SHR) rats than in control SHR rats receiving oil vehicle [235]. It is possible that AVP contributes to the elevation of blood pressure in DOCA-induced hypertension through the augmentation of the neurogenic component of vascular resistance. Studies conducted on DOC-treated and saline-treated rats revealed a significant increase in vascular resistance that was associated with amplification of the central sympathetic tone only in DOC–salt rats. These effects were intensified by vasopressin. Moreover, a reduction in vascular resistance was observed in DOC–salt rats treated with AVP antagonist (I-deaminopenicillamine, 4-valine, 8-D-arginine vasopressin, dPVDAVP) and in those who underwent lumbar sympathectomy [236]. Experiments on dogs maintained on a normal-salt diet showed that a subcutaneous administration of DOC elicited an increase in blood pressure, which was accompanied by an increase in cardiac output, hypernatremia, and an elevation of vasopressin concentration in plasma and in the cerebrospinal fluid (CSF) at the early stage of hypertension [237].

Some evidence suggests that an inappropriate interaction of steroid hormones and vasopressin may play a role in the development of congestive heart failure. For instance, it has been reported that the treatment of sheep with paced-induced heart failure with urocortin 2 (Ucn2—a group of peptides sharing structural similarities with CRH combined with canrenoic acid), which is an antagonist of mineralocorticoid receptors, led to better hemodynamics than the application of canrenoic acid alone. The combined treatment also contributed to reductions in PRA, Ang II, and AVP concentrations, as well as to an improvement of the kidneys’ function [238].

### 4.2. Metabolic Diseases

A growing number of studies address the question of whether interactions between AVP and steroids are altered in metabolic diseases. Clinical data, based on measurements of blood copeptin concentrations, suggest that AVP may play a role in the pathogenesis of the metabolic syndrome (MetS). In this line, obese men with elevated fat free mass and total fat mass show higher fasting glucose and insulin concentrations, as well as an enhanced pituitary response to combined CRH/AVP stimulation. These results suggest that the joint stimulation of CRH and AVP, which is associated with an excessive stimulation of ACTH release, may promote the development of body overweight, adiposity, and hyperinsulinemia [239]. There is also evidence that obesity causes a significant rearrangement of the activation of the HPA by the noradrenergic system. For instance, noradrenergic transporter activity (NAT) assessed in positron emission tomography (PET) was found to correlate differently in obese and non-obese patients. In the non-obese patients, a positive correlation was found between NAT and HPA activity, whereas in the obese patients the correlation was negative. The study suggested that, in the obese patients, the regulation of HPA by the noradrenergic system mainly activates the hypothalamic neurons, whereas in non-obese subjects it engages the prefrontal–limbic cortex more intensively [240]. It has also been postulated that the altered association of copeptin and insulin resistance is related to the elevation of hepatic glycogenolysis; increased insulin, glucagon, and ACTH secretions; and the enhanced activation of 11β-HSD2 [241]. Links between specific steroid hormones and vasopressin in hypoglycemia are less evident. It has been reported that insulin-induced hypoglycemia increases the secretion of cortisol, but it does not affect plasma AVP levels [242]. In another study, more drastic hypoglycemia (1.6 mmol/L), probably producing hypoglycemic stress, caused a rapid significant increase in serum copeptin concentration, which was positively correlated with ACTH and cortisol concentrations. Interestingly, these effects were observed only in women. In men, there was no correlation between copeptin and blood ACTH levels during hypoglycemia and only a poor correlation was found between copeptin and blood cortisol levels during hypoglycemia [243]. Studies conducted on AVP-deficient Brattleboro rats that were also suffering from diabetes mellitus showed that AVP does not play a significant role in the regulation of the release of HPA hormones during acute stress [244].

Balapattabi et al. (2021) examined the associations of liver failure with AVP secretion and hyponatremia in terms of sex differences. They showed that male rats with a bile duct ligation (BDL) were hyponatremic and manifested significantly higher concentrations of plasma copeptin and higher FosB expression in their supraoptic AVP neurons than the sham males, whereas similar associations were not observed in female BDL rats [245]. A study conducted in women with polycystic ovary syndrome revealed that their AVP peak responses to hypoglycemia were negatively correlated with testosterone, androstenedione, and endogenous insulin levels; however, there was no correlation between AVP and basal and hypoglycemia-induced peak cortisol concentrations [246].

Overweight/obese women manifested higher ACTH and cortisol responses to AVP tests and significantly greater hormone inhibition after alprazolam (benzodiazepine used for the treatment of stress and depression) than controls. In both groups, AVP-induced delta-peak cortisol values before and after alprazolam pre-treatment were significantly correlated. Body fat distribution had no effect on the HPA’s response to AVP either before or after alprazolam [247].

## 5. Impact of Therapeutic Interventions on Interactions of Vasopressin with the Hypothalamo–Pituitary–Adrenal System in Health and in Cardiovascular and Metabolic Diseases

As discussed in the previous parts of this review, there are several interconnections between steroids and vasopressin that are significantly affected in cardiovascular, metabolic, and psychogenic disorders [157,168,239]. Thus, it is justified to assume that various therapeutic interventions which have an impact on the vasopressinergic system and the hypothalamo–pituitary–adrenal axis may potently influence the effectiveness of AVP-HPA interactions.

Systemically applied corticosteroids are widely used in the therapy of inflammatory and autoimmune disorders [163,236,248]. Their influence on the HPA and vasopressin secretion resembles the role of endogenous corticosteroids in a negative feedback loop. A post-mortem study by Erkut et al. (1998) on nine patients treated with a corticosteroid and on eight control subjects demonstrated a significant decrease in CRH- and AVP-releasing neurons in the hypothalamus. The study showed that the corticosteroid-treated patients retained only 3.3% of their CRH-releasing cells and 33% of their AVP-releasing cells in comparison to the control group [249]. Thus far, the clinical implications of these changes have not been fully recognized. A study on premature infants with bronchopulmonary dysplasia revealed that treatment with dexamethasone improved their pulmonary functions but did not modify their AVP levels [250]. On the other hand, the administration of corticosteroids in supraphysiological doses in a clinical trial on children with acute lymphoblastic leukemia (ALL) resulted in the suppression of the HPA for a few weeks in the majority of patients [251]. A decrease in AVP mRNA was also found in the SChN of patients treated with corticosteroid (2-fold lower level in the SChN of patients exposed to corticosteroid in comparison to control subjects). Accordingly, it has been proposed that corticosteroid-induced changes in AVP synthesis may account for the circadian rhythm abnormalities and sleep disturbances in steroid-treated patients [252].

**Figure 4:**
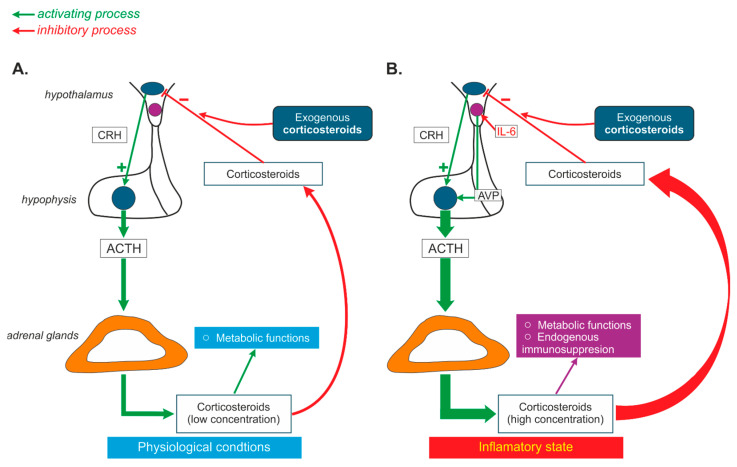
The phenomenon of “corticosteroid escape”. (**A**)—under normal conditions, corticosteroids inhibit the release of corticotrpine-releasing hormone (CRH), (**B**)—during inflammatory states, the activation of autoimmune processes’ and inhibition of CRH secretion via negative feedback are impaired and the activation of the HPA is maintained in spite of the high concentration of corticosteroids. ACTH—adrenocorticotropic hormone, IL-6—interleukin 6, green arrows—stimulation, purple (red) arrows—inhibition.

It has been postulated that vasopressin synthesized in the magnocellular cells of the hypothalamus participates in a phenomenon known as “corticosteroid escape”. According to this hypothesis, inflammatory mediators (for instance IL-6) increase AVP release from hypothalamic magnocellular PVN cells above physiological concentrations, which results in a strong activation of adenylyl cyclases (AC2 and AC7) and an elevation of cAMP to a level at which it can inhibit the negative feedback mediated by endogenous corticosteroids in the HPA. Most likely, the rearrangement of mutual relations between CRH, AVP, interleukins, and corticosteroids accounts for the “corticosteroid escape” that is observed during the activation of the host immune system and that maintains corticosteroid release in spite of the high dose of exogenous corticosteroid imposed by the therapy (Figure 4) [253]. However, the role of “corticosteroid escape” in the achievement of the optimum results from systemic corticosteroid therapy is not yet fully determined.

### 5.1. Steroids and Vasopressin Treatments in Cardiovascular Diseases

It is likely that the resetting of the vasopressin-HPA system by corticosteroids plays a beneficial role in the treatment of severe cardiovascular disorders such as myocardial infarction, shock, and cardiac arrest. A study on the rat model of myocardial infarction revealed that a hydrocortisone administration during the early reperfusion period results in decreased infarct size and in reduced oxidative stress [254]. The potentially beneficial role of corticosteroids may result from their anti-inflammatory activity. There are some concerns regarding the use of corticosteroids in acute myocardial infarction because of their negative effects on wound healing and scar formation. The meta-analysis performed by Giugliano et al. [255] indicated a slight reduction in cardiovascular mortality in patients with acute myocardial infarction treated with corticosteroids. However, it should be noted that this survey was based mainly on studies including small number of patients (less than 100) and it did not equivocally indicate the benefit of treatment with corticosteroids [255]. In another study, the best survival rate (91%) was found in patients with a low baseline cortisol level and with an appropriate adrenal response to ACTH analogs (a difference between minimum and maximum cortisol levels higher than 9 μg/dL) [256]. A study on a cohort of 159 patients with septic shock revealed that the survival rate was significantly greater when vasopressin was used together with hydrocortisone [257,258]. This finding suggested a potential benefit of the joint application of corticosteroids and vasopressin in critically ill patients. More recently, a randomized clinical trial on 512 patients from 10 hospitals in Denmark showed a significant increase in the probability of the return of spontaneous circulation (ROSC) in patients receiving a combined treatment of methylprednisolone and vasopressin in comparison to patients receiving a placebo. However, there was no difference in the 30-day mortality and neurological outcomes of the patients [259].

Studies on overweight/obese women have shown that that obesity is associated with higher ACTH and cortisol responses to AVP tests than those of women with normal body weight, the control, which suggests that obesity may cause the disarrangement of interactions of vasopressin with the HPA [247].

### 5.2. Impact of Anti-Depressive and Neuroleptic Treatments on Vasopressin–HPA Interactions

As was mentioned above, the vasopressin–HPA system is controlled by monoamine neurotransmitters, and the administration of anti-depressive or other neuroleptic compounds, which interfer with monoaminergic transmission, significantly influences AVP-HPA interactions (see Section 3.4).

Moreover, it has been reported that the activation of the HPA during stress seems to be altered in patients with depression and that vasopressin plays a more important role than CRH in the regulation of ACTH release in this group of patients [259,260]. The altered action of vasopressin in patients with depressive and stress-related disorders opens a discussion on the usefulness of selective AVP antagonists as anti-depressive or anxiolytic drugs [261,262]. Undoubtedly, further studies are needed to explain whether the down-regulation of the vasopressinergic pathways would play a beneficial role in these disorders.

A study upon an animal model of depression (olfactory bulbectomy in mice, OB mice) revealed that OB mice demonstrated higher vasopressin and ACTH plasma levels. Moreover, it was possible to reverse theses elevations by chronic treatment with anti-depressive drugs, namely fluoxetine (SSRI) and venlafaxine (SNRI) [261]. Another study on healthy rats showed that antidepressant compounds, such as SSRI and desipramine (tricyclic antidepressant) influence the function of the HPA by means of vasopressin V1bRs [263]. Studies on Wistar rats have shown that the AVP release from magnocellular and parvocellular cells may be affected by neuroleptic drugs and that clozapine and olanzapine are more effective than haloperidol [264].

A study on six patients with atrial fibrillation who qualified for electrical cardioversion and six patients with depression who qualified for electroconvulsive therapy revealed a significantly enhanced activation of their HPA after these interventions. In particular, their AVP concentration increased by 7 times after electrical cardioversion and 2 times after electroconvulsive therapy, whereas their plasma ACTH levels were elevated 3 times and 4 times, respectively [265].

## 6. Summary

**Figure 5:**
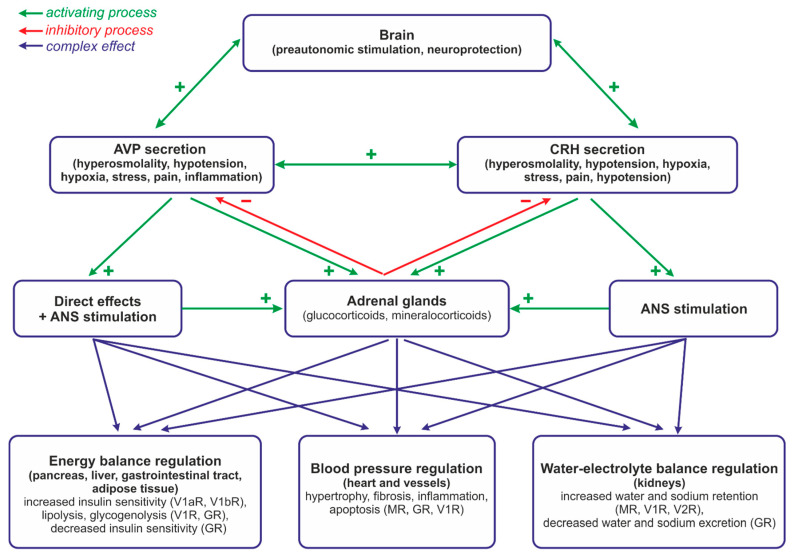
The stimulatory and inhibitory interactions between vasopressin and hormones secreted by the hypothalamo–pituitary–adrenal system that play essential roles in the regulation of energy balance, water–electrolyte balance, and blood pressure. ANS—autonomic nervous system, AVP—arginine vasopressin, GR—glucocorticoid receptor, MR—mineralocorticoids receptor, V1R, V1aR, and V1bR—vasopressin receptors. See text for further explanations.

The present review analyses the complex mechanisms underlying the cooperation of vasopressin with hormones of the hypothalamo–pituitary–adrenal axis. As shown in Figure 5, AVP cooperates with specific components of the HPA through interactions occurring both in the central nervous system and in the peripheral organs.

This cooperation plays a significant role in the regulation of blood circulation, metabolism, water–electrolyte balance, and behavioral adaptations to stress challenges. Vasopressin and components of the HPA closely interact and can be considered a functionally united AVP-HPA system. Growing evidence shows that cardiovascular and metabolic diseases, as well as inflammation and stress, are associated with an inappropriate functioning of the AVP-HPA system, and this question should be taken into consideration when pharmacological treatment is planned. The final action of a specific steroid depends on the concentration of the hormone, the type of stimulated receptors, the number of receptors, and the presence of specific enzymatic pathways in the targeted cell. In the cardiovascular system and in the organs regulating energy balance, stimulations of vasopressin and steroid receptors initiate a wide range of actions whose final effect may be either beneficial or detrimental (Figure 5).

## 7. Future Directions

Our abundant knowledge of the molecular processes initiated by vasopressin and steroid hormones in specific cells and organs is in contrast to our sparse knowledge of their actions in whole organisms. Experimental studies provide strong evidence that the secretion and action of vasopressin and steroid hormones is significantly altered in cardiovascular and metabolic diseases but it is not yet sufficiently understood which mechanisms are responsible for these changes and to what extent the alterations depend on age, sex, the time of application, the presence of other challenges, such as stress and other pathogenic factors, and treatment with specific pharmaceuticals. These denote essential directions for further research in this area.

Moreover, it should be noted that the prevailing number of studies showing the significant interaction of vasopressin and steroids in the regulation of metabolism, cardiovascular functions, and water–electrolyte balance comes from experiments performed on animals, whereas these questions remain largely unexplored in human beings. Undoubtedly, the clinical importance of the findings provided by the animal experiments should be confirmed in studies on large cohorts of human populations, including patients suffering from hypertension, obesity, diabetes mellitus, and atherosclerosis.

## 8. Conclusions

1. Vasopressin (AVP) and steroid hormones are frequently released together and closely cooperate in the regulation of blood pressure, metabolism, water–electrolyte balance, and behavior.
2. Vasopressin interacts with specific components of the hypothalamo–pituitary–adrenal axis in the brain and in several peripheral organs and tissues, including the heart, vessels, kidneys, and adipose tissue.
3. Appropriate interactions of AVP with the HPA are essential for the efficient regulation of water–electrolyte balance, blood pressure, and energy balance, and it is justified to consider vasopressin and the hypothalamo–pituitary axis as a highly coordinated, functional AVP-HPA system.
4. Interactions between AVP and HPA are significantly altered in cardiovascular, respiratory, and metabolic diseases and during inflammation and neurogenic stress.
5. Inappropriate interactions of AVP and steroids may initiate or intensify cardiovascular complications in metabolic diseases.
6. The interplay of vasopressin and steroid hormones is not yet fully recognized and further studies are needed to determine the potentially beneficial or harmful consequences of interference with these factors in the treatment of specific pathological states.

## Data Availability

Not applicable.

## Abbreviations

ACh—acetylcholine, ACTH—adrenocorticotropic hormone, Ang—angiotensin, AR—androgen receptor, AQP—aquaporin, AVP—arginine vasopressin, BDL—bile duct ligation, BNST—bed nucleus of the stria terminalis, cAMP—cyclic adenosine monophosphate, CBI—Coactivator Binding Inhibitor, COVID-19—coronavirus disease 2019, CRF—cortictropin-releasing factor, CRH—corticotropin-releasing hormone, CT—cardiotrophin, CTGF—connective tissue growth factor, DBD—DNA-binding domain, DHEA—dehydroepiandrosterone, DOC—deoxycorticosterone, ERK—extracellular signal-regulated kinase, ENaC—epithelial sodium channel, ES—estrogen, ESR—estrogen receptor, FSH—follicle-stimulating hormone, GABA—gamma aminobutyric acid, GC—glucocorticoid, GnRH—gonadotropine-releasing hormone, GP—glycopeptin, copeptine, GPER—G-protein-coupled receptor, GR—glucocorticoid receptor, GRK2—G-protein-coupled receptor kinase 2, HDL—high-density lipoprotein, HIF—hypoxia-inducible factor, HSD—hydroxysteroid dehydrogenase, ICAM—intercellular adhesion molecule, IL—interleukin, JNK—Jun N-terminal kinase, LBD—ligand-binding domain, LDL—low-density lipoprotein, LH—luteinizing hormone, MAPK—mitogen-activated protein kinase, MC—mineralocorticoid, MCP-1—monocyte chemoattractant protein-1, MCR, MR—mineralocorticoid receptor, MRE—mineralocorticoid response element, NADPH—dinicotinamide adenine dinucleotide phosphate, NAT—noradrenergic transporter activity, NCOAS—nuclear receptor co-activator, NCX—sodium–calcium exchanger, NFκB—nuclear factor kappa B, NO—nitric oxide, NOX—nicotinamide adenine dinucleotide phosphate oxidase, NP—neurophysin, NR3C2—nuclear receptor of subfamily 3, group C, member 2, NTD—N-terminal domain, NST—nucleus of the solitary tract, OVLT—organum vasculosum of the lamina terminalis, PET—positron emission tomography, PG—progesterone, PGR—progesterone receptor, PKA—protein kinase A, PKC—ptotein kinase C, PLC—phospholipase C, PPAR—peroxisome proliferator-activator receptor, PVN—parventricular nucleus, RAS—renin angiotensin system, ROS—reactive oxygen species, ROSC—return of spontaneous circulation, SFO—subfornical organ, SHR—spontaneously hypertensive rat, SON—supraoptic nucleus, SNP—single nucleotide polymorphism, SRC—steroid receptor co-activator, TNF-α—tumor necrosis factor alpha, UCMS—unpredictable chronic mild stress, UCP—uncoupling protein, UT—urea transporter, V1aR—vasopressin receptor of type V1a, V1bR—vasopressin receptor of type V1b, V2R—V2 vasopressin receptor of type 2, VS—vasopressin system.
